# Implementation of the cervical cancer prevention policy in one of the regions of Lithuania

**DOI:** 10.18332/ejm/192520

**Published:** 2024-09-30

**Authors:** Gerda Bukauskaitė Žiūkienė, Alina Liepinaitienė

**Affiliations:** 1 Department of Nursing, Faculty of Medicine, Kauno kolegija Higher Education Institution, Kaunas, Lithuania

**Keywords:** cervical cancer, prevention, vaccination, HPV

## Abstract

**INTRODUCTION:**

Although the Lithuanian government increases funding for the cervical cancer prevention program every year, the incidence and mortality rates of cervical cancer are among the highest in Europe. In order to improve the prevention policy regarding cervical cancer, it is necessary to investigate the implementation of the cervical cancer prevention policy in one of the regions in Lithuania.

**METHODS:**

A quantitative survey method using a questionnaire, was applied in one of the regions of Lithuania. The study was conducted in the period 1–18 April 2022. During the study, 213 residents of the investigating region were interviewed.

**RESULTS:**

Respondents evaluated the cervical cancer prevention program in the investigated region positively, but not all women received invitations to participate in this program. The research revealed that the residents of the city of investigation have received this invitation more often than the women living in other districts.

**CONCLUSIONS:**

Women’s opinion about the effectiveness of the cervical cancer prevention program is positive. Still, there is an emphasis on the wish that this program could be used from an the age of 25 years and continue to 59 years. The prevention program could be carried out more often than is currently established.

## INTRODUCTION

Cervical cancer begins in the cells of the cervix. A cancerous (malignant) tumor is a group of cancerous cells that can grow into and destroy nearby tissue^1^. Changes in the cells of the cervix can also cause precancerous conditions. This means that the abnormal cells are not yet cancerous, but there is a chance that they could become cancerous if left untreated for a long time^2,3^. The most common precancerous condition of the cervix goes by different names depending on how it is classified or reported^4^. The most common classifications of precancerous cervical conditions are squamous intraepithelial disease (SIL), cervical intraepithelial neoplasia (CIN), and cervical dysplasia. Cervical cancer is the fourth most common cancer in women worldwide and the second most common in the European Union. Only in Europe in 2018, 61000 new cases and 25000 deaths were reported, despite cervical cancer being preventable when detected in its precancerous state. On 19 May 2023, the Director-General of WHO issued a call for action to reduce cervical cancer and called for services to be integrated into strong health systems to ensure universal health coverage^5^.

Many European countries have implemented organized cervical screening, and about 70% of EU citizens have the opportunity to participate in an organized program. Their success is clear: in countries with an organized screening program, morbidity and mortality have decreased by up to 70%^6^. Nevertheless, cervical cancer is still common and even increasing in several Central and Eastern European countries. Other parts of Europe are also reporting an increasing incidence. For example, cervical cancer rates have increased by 15% to 30% in the past decade in the Netherlands, Sweden, and the UK. This highlights the need for more effective implementation of preventive measures to prevent cervical cancer from becoming a public health problem^7^.

In Lithuania, the number of cases of cervical cancer is increasing every year, and the mortality rate from this disease is one of the highest in Europe. Although the government increases funding for the cervical cancer prevention program every year, the incidence and mortality rates of cervical cancer are among the highest in Europe^8^. There is limited information on cervical cancer policy in Lithuania; one study analyzed family doctors’ opinions and personal experiences in implementing the cervical cancer prevention program and compared the attitudes of younger and older family doctors working in the public and private healthcare sectors to this prevention program^9^. Another analyzed the management of the cervical cancer prevention program in the context of Lithuania’s protection policy, reviewing the cervical cancer prevention program, its management, and its improvement^10^.

Although a cervical cancer prevention program has been implemented in Lithuania since 2004, about 400 new cases of cervical cancer are still diagnosed in the country every year, and up to 200 deaths are registered per year^2,3^. The study aims to investigate the implementation of the cervical cancer prevention policy in one of the regions in Lithuania.

## METHODS

### Study design and collection of data

To review the implementation of the cervical cancer prevention program in one of the regions in Lithuania, a quantitative study using a cross-sectional design and anonymous questionnaires was conducted during 1–18 April 2022.

### Participants

A total of 31415 women aged 25–59 years lived in the studied region in 2020. In order to survey this general population with at least 7% error, it was estimated that at least 203 women in the study region were needed to be interviewed^2^. The sampling process was community-based. Women were invited to participate in the study by receiving an open invitation to the survey using a convenience sampling method.

The participants who agreed to participate in the research were willing to share information, were sincere, tried to give their answers as clearly as possible, and sought mutual understanding. All women gave informed consent to participate in the survey.

The questionnaire survey included information about women’s knowledge and opinions about cervical cancer screening programs in Lithuania, and what kind of information they are missing from the healthcare givers.

### Ethics

In order not to violate the research ethics, when placing the research questionnaire in the groups, the purpose and nature of the research and the use of the received answers were briefly explained. The introductory part of the questionnaire also contained written information about the research being conducted. It clearly described how to complete the questionnaire and who can participate (selection criteria are indicated). It emphasized that filling in the questionnaire was not mandatory and can be stopped at any time by free will. Anonymity was maintained throughout the research. The aggregated results were used for study purposes only. Subjects were not required to provide their contact details or information that would reveal their identity. Ethical approval was obtained from Mykolas Romeris University (No. 20-11-2019).

### Statistical analysis

Descriptive statistics were used to assess the distribution of the characteristics under consideration in the selected sample using frequencies and percentages. In order to compare the results of the study according to the age of women, we divided all subjects into two groups based on the median age of 37.5 years: those aged ≤37 years and those aged ≥38 years. Tables of related characteristics were created to evaluate the relationship of characteristics, the chi-squared (χ^2^) criterion was calculated to determine the dependence of characteristics, and equality of proportions was assessed by z-test with Bonferroni correction. After calculating the chi-squared and comparing the proportions with the help of z-tests, statistically significant (p<0.05) differences were determined.

## RESULTS

During the study, 213 residents of the study region were interviewed, whose average age was 37.8 ± 9.3 years. Most women (66%) rated their health as very good and good, while only 1% rated it as poor. Results showed that women aged ≤37 years rated their health as very good significantly more often compared to those aged ≥38 years, while married or partnered women rated their health significantly more often than single women. Meanwhile, health assessments according to other sociodemographic characteristics did not differ statistically significantly (p>0.05), as noted in Table 1.

**Table 1:** Distribution of self-reported current health by sociodemographic characteristics of women aged 25–59 years, 1–18 April 2022, Lithuania (N=213)

*Characteristics*		*Current health*				*χ^2^*	*df*	*p*
		*Very good n (%)*	*Good n (%)*	*Mediocre n (%)*	*Weak n (%)*			
**Age** (years)	≤37	15 (14.2)*	59 (55.7)	30 (28.3)	2 (1.9)	**8.46**	**3**	**0.037**
	≥38	5 (4.7)	61 (57.5)	40 (37.7)	0 (0)			
**Residence**	City	15 (11.2)	73 (54.5)	45 (33.6)	1 (0.7)	1.72	3	0.633
	Suburb	5 (7.0)	44 (62.0)	21 (29.6)	2 (1.4)			
**Marital status**	Single	2 (6.5)	12 (38.7)	16 (51.6)	1 (3.2)	**7.99**	**3**	**0.046**
	Married	18 (9.9)	108 (59.3)**	55 (30.2)	1 (0.5)			
**Education level**	Lower than Higher	3 (4.9)	30 (49.2)	27 (44.3)	1 (1.6)	6.11	3	0.107
	Higher	17 (11.2)	90 (59.2)	44 (28.9)	1 (0.7)			

Statistically significant (p<0.05) differences are in bold:

* p<0.05, compared to women aged ≥38 years;

** p<0.05, compared to single women.

The most commonly mentioned factors influencing the development of cervical cancer included the human papilloma virus (HPV) and a weakened immune system while sexually transmitted infections were less commonly reported. The most commonly mentioned factors influencing the development of cervical cancer were compared according to women’s sociodemographic characteristics. Statistically significant (p<0.05) differences were found, which showed that sexually transmitted diseases, smoking, and the use of contraceptive pills were statistically significantly more often mentioned by women aged ≤37 years.

Human papillomavirus and sexually transmitted diseases were named significantly more often by women with higher education compared to women with primary, secondary, and special education, as noted in Table 2.

**Table 2:** The most frequently mentioned factors affecting the development of cervical cancer according to sociodemographic characteristics of women aged 25–59 years, 1–18 April 2022, Lithuania (N=213)

*Characteristics*		*Human papilloma virus (HPV) n (%)*	*Weakened immune system n (%)*	*Sexually transmitted diseases n (%)*	*Smoking n (%)*	*Contraceptive pills n (%)*
**Age** (years)	≤37	86 (81.1)	60 (56.6)	61 (57.5)*	42 (39.6)*	30 (28.3)*
	≥38	84 (79.2)	55 (51.9)	37 (34.9)	23 (21.7)	15 (14.2)
	p	0.730	0.491	**0.001**	**0.005**	**0.012**
**Residence**	City	105 (78.4)	75 (56.0)	63 (47.0)	44 (32.8)	27 (20.1)
	Suburb	60 (84.5)	38 (53.5)	31 (43.7)	20 (28.2)	15 (21.1)
	p	0.291	0.737	0.647	0.493	0.869
**Marital status**	Single	23 (74.2)	17 (54.8)	11 (35.5)	7 (22.6)	7 (22.6)
	Married	147 (80.8)	99 (54.4)	87 (47.8)	58 (31.9)	38 (20.9)
	p	0.399	0.963	0.203	0.299	0.830
**Education level**	Lower than Higher	40 (65.6)	30 (49.2)	19 (31.1)	14 (23.0)	10 (16.4)
	Higher	130 (85.5)**	86 (56.6)	79 (52.0)**	51 (33.6)	35 (23.0)
	p	**0.001**	0.327	**0.006**	0.129	0.284

Statistically significant (p<0.05) differences are in bold:

* p<0.05, compared to women aged ≥38 years;

** p<0.05, compared to women with lower than higher education.

Participation in a cervical cancer prevention program was compared according to women’s sociodemographic characteristics. Statistically significant differences (p<0.05) were determined, which showed that significantly more women aged ≥38 years participated in the program compared to those aged ≤37 years. Meanwhile, participation in the program according to other sociodemographic characteristics did not differ statistically significantly (p>0.05), as noted in Table 3.

**Table 3:** Distribution of participation in a cervical cancer prevention program by sociodemographic characteristics of women aged 25–59 years, 1–18 April 2022, Lithuania (N=213)

*Characteristics*		*Participation in the cervical cancer prevention program*				*χ^2^*	*df*	*p*
		*Yes*		*No*				
		*n*	*%*	*n*	*%*
**Age** (years)	≤37	74	69.8	32	30.2	**12.63**	**1**	**0.000**
	≥38	94*	**89.5**	11	10.5			
**Residence**	City	110	82.7	23	17.3	0.82	1	0.364
	Suburb	55	77.5	16	22.5			
**Marital status**	Single	26	83.9	5	16.1	0.47	1	0.492
	Married	142	78.5	39	21.5			
**Education level**	Lower than Higher	49	80.3	12	19.7	0.06	1	0.805
	Higher	119	78.8	32	21.2			

Statistically significant (p<0.05) differences are in bold;

* p<0.05, compared to women aged ≤37 years.

## DISCUSSION

The findings of this study shed light on several important aspects related to women’s health perceptions, factors influencing cervical cancer development, and participation in cervical cancer prevention programs^11^.

Analyzing the results, it is evident that most women rated their health positively. This trend is consistent with existing literature emphasizing the importance of subjective health assessments in understanding overall well-being. However, significant differences emerged when examining health ratings based on sociodemographic characteristics. Notably, younger women, specifically those aged ≤37 years, were more likely to rate their health as very good than their older counterparts. Similarly, married or partnered women reported better health status than single women. These findings underscore the influence of age and marital status on subjective health perceptions among women. Such insights are crucial for tailoring health interventions and promoting well-being across different sociodemographic groups^12^.

Regarding factors influencing cervical cancer development, the study identified several key determinants, with human papillomavirus (HPV) and weakened immune systems being the most commonly mentioned^13,14^. Conversely, factors such as smoking and use of contraceptive pills were less frequently cited. Furthermore, significant associations were observed between the mention of certain factors and women’s sociodemographic characteristics.

For instance, younger women were more likely to mention sexually transmitted diseases, smoking, and use of contraceptive pills compared to older women. Additionally, women with higher education level were more inclined to cite HPV and sexually transmitted diseases as influential factors. These findings underscore the importance of education and age in shaping awareness of cervical cancer risk factors.

The study also assessed participation in cervical cancer prevention programs, revealing that a majority of women took part in such initiatives. However, significant differences were noted based on age, with older women showing higher participation rates than their younger counterparts. These findings highlight the need for targeted outreach efforts to engage younger women in preventive healthcare initiatives^15^.

Overall, the results provide valuable insights into women’s health perceptions, awareness of cervical cancer risk factors, and participation in prevention programs. By understanding these dynamics, healthcare practitioners and policymakers can develop tailored interventions to promote women’s health and reduce the burden of cervical cancer in the population. Further research is warranted to explore additional factors influencing health behaviors and outcomes among diverse sociodemographic groups.

### Limitations

This work seeks to set baseline knowledge that can be used in cervical cancer prevention programs in Lithuania. However, as a convenience sample was used, the participants may not reflect the general population of Lithuania, while subgroups, such as women who suffer from illnesses were not included in this population. Also, an additional limitation is that the research was performed in one Lithuanian region and hence may not reflect the overall population. Further research with larger sample sizes and a broader geographical area are needed.

## CONCLUSIONS

Over three-quarters of the women who filled in the questionnaires had participated in a cervical cancer prevention program. Still, only one-quarter of the women knew about the latest changes in the prevention program law. The majority noted that they knew that preventive screening for cervical cancer is one of the prevention measures, but only two-thirds of those who filled in the questionnaires marked vaccinations as a method of prevention. Further research and informational interventions on cervical cancer are needed.

## Data Availability

The data supporting this research are available from the authors on reasonable request.

## References

[1] Cohen PA, Jhingran A, Oaknin A, Denny L. Cervical cancer. Lancet. 2019;393(10167):169-182. doi:10.1016/S0140-6736(18)32470-X 30638582

[2] Buskwofie A, David-West G, Clare CA. A Review of Cervical Cancer: Incidence and Disparities. J Natl Med Assoc. 2020;112(2):229-232. doi:10.1016/j.jnma.2020.03.002 32278478

[3] Zhang S, Xu H, Zhang L, Qiao Y. Cervical cancer: Epidemiology, risk factors and screening. Chin J Cancer Res. 2020;32(6):720-728. doi:10.21147/j.issn.1000-9604.2020.06.05 33446995 PMC7797226

[4] Okunade KS. Human papillomavirus and cervical cancer. J Obstet Gynaecol. 2020;40(5):602-608. doi:10.1080/01443615.2019.1634030 31500479 PMC7062568

[5] Miller AB. Cervix cancer. In: Kramer BS, Gohagan JK, Prorok PC, eds. Cancer screening. (pp. 195-217). CRC Press; 1999:195-217.

[6] Ferrall L, Lin KY, Roden RBS, Hung CF, Wu TC. Cervical Cancer Immunotherapy: Facts and Hopes. Clin Cancer Res. 2021;27(18):4953-4973. doi:10.1158/1078-0432.CCR-20-2833 33888488 PMC8448896

[7] Hull R, Mbele M, Makhafola T, et al. Cervical cancer in low and middle-income countries. Oncol Lett. 2020;20(3):2058-2074. doi:10.3892/ol.2020.11754 32782524 PMC7400218

[8] Kojalo U, Tisler A, Parna K, et al. An overview of cervical cancer epidemiology and prevention in the Baltic States. BMC Public Health. 2023;23(1):660. doi:10.1186/s12889-023-15524-y 37029357 PMC10080753

[9] Everatt R, Kuzmickienė I, Intaitė B, Anttila A. Effectiveness of the cervical cancer prevention programme: a case-control mortality audit in Lithuania. Eur J Cancer Prev. 2020;29(6):504-510. doi:10.1097/CEJ.0000000000000603 32932287

[10] Kuciauskaite G, Jariene K, Kvitkovskaja K. An audit of colposcopy cases for cervical cancer prevention in Lithuanian hospitals. Eur J Obstet Gynecol Reprod Biol. 2024;293:102. doi:10.1016/j.ejogrb.2023.08.286

[11] Bedell SL, Goldstein LS, Goldstein AR, Goldstein AT. Cervical Cancer Screening: Past, Present, and Future. Sex Med Rev. 2020;8(1):28-37. doi:10.1016/j.sxmr.2019.09.005 31791846

[12] Canfell K, Kim JJ, Brisson M, et al. Mortality impact of achieving WHO cervical cancer elimination targets: a comparative modelling analysis in 78 low-income and lower-middle-income countries. Lancet. 2020;395(10224):591-603. doi:10.1016/S0140-6736(20)30157-4 32007142 PMC7043006

[13] Razali N, Mostafa SA, Mustapha A, Abd Wahab MH, Ibrahim NA. Risk factors of cervical cancer using classification in data mining. J Phys Conf Ser. 2020;1529(2):022102. doi:10.1088/1742-6596/1529/2/022102

[14] Elshami M, Thalji M, Abukmail H, et al. Knowledge of cervical cancer risk factors among Palestinian women: a national cross-sectional study. BMC Womens Health. 2021;21(1):385. doi:10.1186/s12905-021-01510-2 34727914 PMC8561913

[15] Nagendiram A, Bougher H, Banks J, Hall L, Heal C. Australian women’s self-perceived barriers to participation in cervical cancer screening: A systematic review. Health Promot J Austr. 2020;31(3):343-353. doi:10.1002/hpja.280 31353682

